# Evidence of the specific roles of autophagy in senescent leaves and maturing seeds

**DOI:** 10.1080/27694127.2025.2472160

**Published:** 2025-03-07

**Authors:** Anne Marmagne, Fabien Chardon, Céline Masclaux-Daubresse

**Affiliations:** Université Paris-Saclay, INRAE, AgroParisTech, Institute Jean-Pierre Bourgin for Plant Sciences (IJPB), France

**Keywords:** Arabidopsis, nitrogen remobilization, seed filling, source leaves, sink tissues

## Abstract

In plants, a large part of the nutrients used to generate seed lipid and protein reserves is derived from both the degradation of macromolecules in source leaves and the transfer of small catabolic molecules like amino acids from the senescing leaves to the seeds. Studies of autophagy mutants in *Arabidops*is showed that autophagy is a master player controlling 60% of the remobilization of nitrogen from senescing leaf tissues to developing seeds, and strongly impacting reserve deposition, especially in the protein to lipid ratio. Since autophagy is largely enhanced in leaves during senescence and in the seeds during maturation, we investigated the roles of autophagy in these sources and sink tissues, to identify checkpoints controlling seed filling and quality. Through gene complementation using tissue-specific promoters, we demonstrated that while autophagy regulates nitrogen flux to the seeds in source leaves, the autophagy taking place in seeds during their maturation is essential to reach the appropriate seed quality in terms of C and N storage. Overall, these results highlight the multiple roles of autophagy in the optimal development of the plant throughout its entire lifespan

## TEXT

In plants, transcriptomic data have revealed a strong induction of autophagy genes during rosette leaf senescence and seed maturation. The induction of autophagy during these two critical stages in the plant’s life underscores its essential role in the source-sink relationship. In previous studies investigating autophagy mutants, we uncovered important roles of autophagy in both the remobilization of nitrogen to seeds and efficient seed filling. However, the experimental design made it difficult to determine whether the observed defects in seed filling and nitrogen remobilization were caused by impaired autophagy within either the seed or the source leaves.

In our recent manuscript [1], we provided evidence for the specific roles of autophagy in both source and sink organs by using organ-specific expression constructs in the *Arabidopsis atg5 (autophagy-related 5)* autophagy mutant. While to restore autophagy activity in the source leaves, we chose to express the ATG5 wild-type allele under the promoter of the *SAG12* (senescence-associated gene 12), we opted for the soybean *Gly1 (Glycinin-1*) promoter for the seeds under maturation. Expression in both organs was achieved by co-expressing the two constructs in the *atg5* mutant.

**Figure 1.**
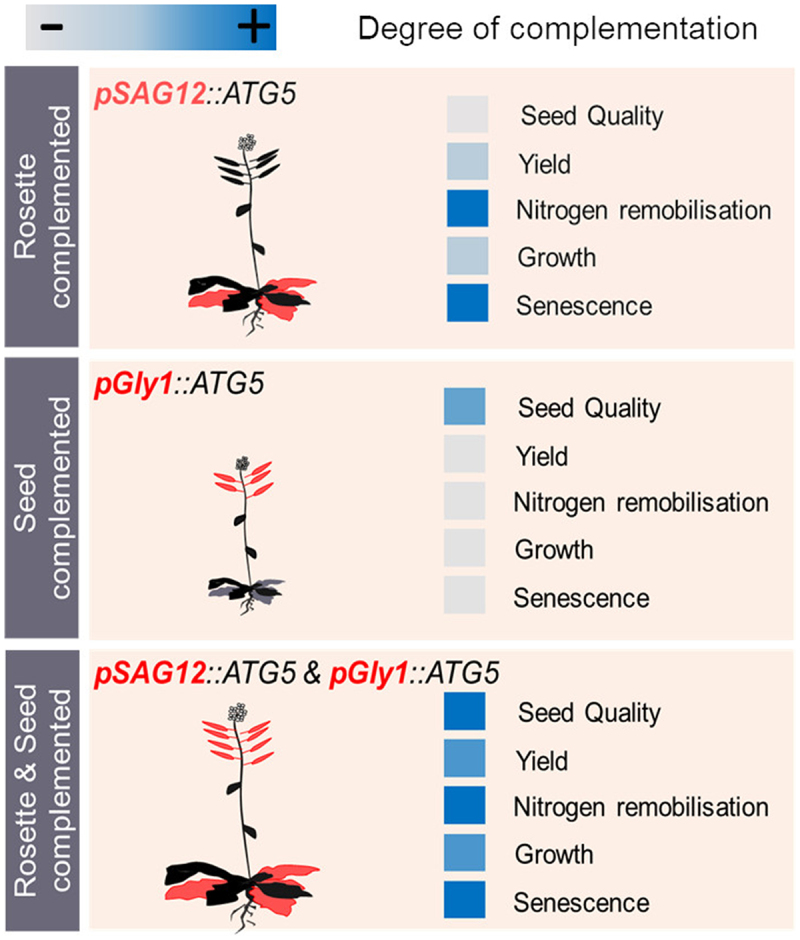
Summary of the effects of different *ATG5* tissue-specific complementations in the Arabidopsis *atg5* mutant. The *pSAG12* promoter targets complementation to senescing leaves. The *pGly1* promoter targets expression to the seed embryos during maturation. ATG5 expression sites are shown in red. Blue squares indicate for each trait the recovery levels (from low recovery (light blue) to wild type like recovery (dark blue)) attained after complementation.

Mutation in *Arabidopsis ATG5*, resulting in *atg5* mutants, akin mutations within any single gene of the autophagy core machinery, disrupts the formation of autophagosomes, and leads to plant hypersensitivity to N and C starvations, premature leaf yellowing, and lower plant biomass and seed yield. Our previous studies on nitrogen use efficiency in plants show that these phenotypes are associated with extremely disturbed nitrogen content in all the plant organs. The senescent-leaves complementation *pSAG12::ATG5* rescued the early leaf senescence phenotype of the *atg5* plant (Figure 1). This rescue, characterized by a return of greenness and an increase in chlorophyll content, was associated with a partial increase in both biomass and seed yield. In contrast, the seed-specific complementation *pGly1::ATG5* did not altered the leaf yellowing, biomass or yield, which remained as in the *atg5* mutant. The dual complementation *pGly1::ATG5* and *pSAG12::ATG5* significantly decreased leaf senescence and improved both plant growth and biomass distribution. While rosette-specific complementation *pSAG12::ATG5* fully restored the wild-type biomass allocation across vegetative and reproductive organs, the seed-specific complemented lines *pGly1::ATG5* displayed an intermediate phenotype between *atg5* and the wild type plant. Interestingly, the dual complemented lines exhibited even better growth and yield than the rosette-specific complemented lines, ultimately achieving a biomass distribution comparable to that of wild-type *Arabidopsis*.

In addition, our examination of nitrogen remobilisation using ^15^N-labeled nitrate revealed that expression of *ATG5* under the control of rosette-specific promoter *pSAG12*, but not under the seed-specific promoter *pGly1*, significantly enhanced nitrogen flux from rosette leaves to seeds, restoring N remobilization to wild type levels. While the rosette-specific complementation *pSAG12::ATG5* successfully improved the nitrogen status of rosette and stem organs, it did not affect nitrogen concentrations in seeds. In contrast, the seed-specific complementation *pGly1::ATG5* was completely ineffective in restoring nitrogen concentrations in rosette, stem and siliques, although it partially recovered nitrogen and carbon concentrations in seeds. Remarkably, only the dual complementation pSAG12::ATG5 plus pGly1::ATG5 fully restored seed filling to wild type level.

In summary, our investigation underscores the pivotal role of autophagy in both senescing leaves and in seed embryos. In aging leaves, enhanced autophagy is essential for the stepwise degradation and recycling of macromolecules to ensure phloem loading and efficient remobilization of nitrogen and other nutrients as sulphur, zinc or iron, to the seeds. However, our results reveal that while autophagic activity in senescent leaves supports nitrogen remobilisation, successful seed filling and proper carbon and nitrogen storage also require active autophagy in seed embryos during maturation. Autophagic activity in seeds at the maturation stage acts as a crucial checkpoint, ensuring optimal seed filling and adequate nutrient storage. The absence of autophagy in seeds leads to imbalances in carbon and nitrogen concentrations, disrupts lipid and protein biosynthesis and ultimately compromises seed quality.

## ABBREVIATIONS

ATG5: autophagy-related 5
Gly1: Glycinin-1
*SAG12*: senescence-associated gene 12
pGly1: Glycinin-1 promoter
p*SAG12*: senescence-associated gene 12 promoter
